# Amentoflavone as an Ally in the Treatment of Cutaneous Leishmaniasis: Analysis of Its Antioxidant/Prooxidant Mechanisms

**DOI:** 10.3389/fcimb.2021.615814

**Published:** 2021-02-25

**Authors:** Yasmin Silva Rizk, Sandy Santos-Pereira, Luiza Gervazoni, Daiana de Jesus Hardoim, Flávia de Oliveira Cardoso, Celeste da Silva Freitas de Souza, Marcelo Pelajo-Machado, Carlos Alexandre Carollo, Carla Cardozo Pinto de Arruda, Elmo Eduardo Almeida-Amaral, Tânia Zaverucha-do-Valle, Kátia da Silva Calabrese

**Affiliations:** ^1^ Laboratório de Imunomodulação e Protozoologia, Instituto Oswaldo Cruz, Fundação Oswaldo Cruz, Rio de Janeiro, Brazil; ^2^ Laboratório de Bioquímica de Tripanosomatídeos, Instituto Oswaldo Cruz, Fundação Oswaldo Cruz, Rio de Janeiro, Brazil; ^3^ Laboratório de Patologia, Instituto Oswaldo Cruz, Fundação Oswaldo Cruz, Rio de Janeiro, Brazil; ^4^ National Institute of Science and Technology on Neuroimmunomodulation, Oswaldo Cruz Institute, Oswaldo Cruz Foundation, Rio de Janeiro, Brazil; ^5^ Laboratório de Produtos Naturais e Espectrometria de Massas, Instituto de Biociências, Universidade Federal de Mato Grosso do Sul, Campo Grande, Brazil; ^6^ Laboratório de Parasitologia Humana, Instituto de Biociências, Universidade Federal de Mato Grosso do Sul, Campo Grande, Brazil

**Keywords:** amentoflavone, biflavonoid, prooxidant, antileishmanial activity, intralesional treatment, cutaneous leishmaniasis, Glucantime

## Abstract

Treatment of leishmaniasis is a challenging subject. Although available, chemotherapy is limited, presenting toxicity and adverse effects. New drugs with antileishmanial activity are being investigated, such as antiparasitic compounds derived from plants. In this work, we investigated the antileishmanial activity of the biflavonoid amentoflavone on the protozoan *Leishmania amazonensis*. Although the antileishmanial activity of amentoflavone has already been reported *in vitro*, the mechanisms involved in the parasite death, as well as its action *in vivo*, remain unknown. Amentoflavone demonstrated activity on intracellular amastigotes in macrophages obtained from BALB/c mice (IC_50_ 2.3 ± 0.93 μM). No cytotoxicity was observed and the selectivity index was estimated as greater than 10. Using BALB/c mice infected with *L. amazonensis* we verified the effect of an intralesional treatment with amentoflavone (0.05 mg/kg/dose, in a total of 5 doses every 4 days). Parasite quantification demonstrated that amentoflavone reduced the parasite load in treated footpads (46.3% reduction by limiting dilution assay and 56.5% reduction by Real Time Polymerase Chain Reaction). Amentoflavone decreased the nitric oxide production in peritoneal macrophages obtained from treated animals. The treatment also increased the expression of ferritin and decreased iNOS expression at the site of infection. Furthemore, it increased the production of ROS in peritoneal macrophages infected *in vitro*. The increase of ROS *in vitro*, associated with the reduction of NO and iNOS expression *in vivo*, points to the antioxidant/prooxidant potential of amentoflavone, which may play an important role in the balance between inflammatory and anti-inflammatory patterns at the infection site. Taken together these results suggest that amentoflavone has the potential to be used in the treatment of cutaneous leishmaniasis, working as an ally in the control and development of the lesion.

## Introduction

Leishmaniases are a complex of infectious diseases caused by several species of protozoa from the genus *Leishmania*, which are transmitted between vertebrate and invertebrate hosts by the bite of infected female sandflies. The clinical manifestations range from more severe forms such as visceral leishmaniasis (VL) and mucous leishmaniasis (ML) to more common and benign forms as cutaneous leishmaniasis (CL). In general, the form and severity of the disease depends on the infecting species combined with the host genetics and the immune response generated by the host (Colmenares et al., 2002). With a wide global distribution, leishmaniases put at risk more than one billion people living in endemic areas. It is estimated that 30,000 new cases of visceral leishmaniasis and more than one million cases of cutaneous leishmaniasis occur annually (World Health Organization, 2020).

Pentavalent antimonials and amphotericin B are the most widely used drugs for the treatment of leishmaniases. They have important limitations such as high toxicity, adverse effects and parenteral administration. To further aggravate the current scenario of leishmaniases chemotherapy, treatment varies in effectiveness as a result of the variation in the intrinsic sensitivity of the different *Leishmania* species, acquired resistance and differences in the host immune response (Croft and Coombs, 2003). In 2010, the World Health Organization (WHO) promoted the use of local therapies to treat cases of uncomplicated CL (World Health Organization, 2010) and the intralesional approach was included in the CL treatment recommendations in Brazil (Brasil, 2017), after studies in the country that had already shown the benefits of CL local treatment (Oliveira-Neto et al., 1997; de Oliveira Duque et al., 2016). These studies highlighted not only the decrease in adverse effects and toxicity, but also the cost and accessibility of treatment, especially in rural areas (Añez et al., 2018; Arboleda et al., 2019; de Oliveira Duque et al., 2019). Therefore, intralesional treatment can offer advantages such as simplicity, efficiency, and safety, especially when the patient conditions do not allow systemic chemotherapy (de Oliveira Duque et al., 2019).

In the search for novel and effective treatments to combat leishmaniases, natural products have been used. In addition to exhibiting potential as therapeutic compounds, natural products may also contribute to the development of new drugs based on their chemical structures (Gervazoni et al., 2020). The biflavonoid amentoflavone (3’, 8’’ - Biapigenin) is an apigenin dimer linked by a C3’-C8’’ covalent bond. Many biological amentoflavone activities have been described, both *in vitro* and *in vivo*.

Several activities and effects have been described for this flavonoid, including a number of studies investigating its anti-inflammatory (Cao et al., 2017), antioxidant (Saroni Arwa et al., 2015; Su et al., 2019), anti-tumor (Yen et al., 2018; Hsu et al., 2019), neuroprotective (Chen et al., 2018) and, recently, anti-SARS-CoV-2 (Mishra et al., 2020) properties. Antileishmanial activity has also been investigated. The biflavonoid was active against promastigotes (Njock et al., 2017) and in a previous study, our group showed that amentoflavone has a leishmanicidal action on intracellular forms, independent of nitric oxide (NO) production (Rizk et al., 2014).

In this study, we evaluated the leishmanicidal activity of amentoflavone *in vivo*, using the intralesional route after BALB/c mice infection with *Leishmania amazonensis* promastigotes, and searched for its mechanism of action. Intralesional treatment with amentoflavone showed no toxicity and reduced the parasitic burden in infected animals, revealing its potential use in the treatment of cutaneous leishmaniasis. Furthemore, amentoflavone treatment increased reactive oxygen species (ROS) production in murine macrophages, consequently reducing the number of intracellular amastigote forms. These results points for the amentoflavone prooxidant effect as its mechanism of action against *Leishmania* sp infection.

## Materials and Methods

### Test Compounds

Amentoflavone was purchased from Sigma-Aldrich^®^ (HPLC grade ≥ 98%). A stock solution was prepared in DMSO (Sigma-Aldrich) and then diluted in the culture medium to obtain the concentrations used in the *in vitro* assays. For the *in vivo* experiments, amentoflavone was solubilized directly in the administration vehicle, as follows: 10% Ethanol + 10% Kolliphor^®^EL (Sigma-Aldrich) + 1% DMSO in phosphate saline (PBS Buffer) pH 7.2. The N-methyl glucamine (Glucantime^®^, Sanofi-Aventis), was kindly provided by the pharmacy of the National Institute of Infectology/FIOCRUZ, Rio de Janeiro, and diluted in PBS when necessary.

### Parasites

Amastigotes of *L. amazonensis* (IFLA/BR/1967/PH8) were isolated from cutaneous lesions of BALB/c mice and maintained as promastigotes at 26°C in liquid medium LIBHIT (Costa and Lagrande, 1981). The medium was supplemented with 10% Fetal Bovine Serum (FBS, Gibco), 10,000 U/L penicillin and 10 mg/L streptomycin (Sigma-Aldrich). Promastigote forms grown in axenic culture were used for infection. Fresh cultures were unfreezed every six passages and always used in the stationary phase (fifth day of growth) to ensure infectivity.

### Intracellular Amastigote Activity

Thioglycollate-elicited peritoneal macrophages from BALB/c mice were seeded in 24-well plates containing coverslips at a concentration of 2 × 10^5^ cells/well in complete RPMI medium - RPMI-1640 medium (Sigma-Aldrich) supplemented with 10% FBS (Gibco), 200 mM L-glutamine, 10,000 U/L penicillin and 10 mg/L streptomycin (Sigma-Aldrich). After adhesion, plates were washed with PBS to remove non-adherent cells and kept overnight at 34°C /5% CO2. Subsequently, macrophages were infected with promastigote forms of *L. amazonensis* 10:1 (parasite/macrophage) in the stationary growth phase for 6 h, and then the culture was washed with PBS pH 7.0 to remove promastigotes from the supernatant. After that, different concentrations of amentoflavone (0–11.14 μM) were added to wells containing infected macrophages, and plates were incubated at 37°C/5% CO_2_ for 72 h. After Bouin-fixing and Giemsa-staining, cells were analyzed by light microscopy. The number of intracellular amastigote forms was determined in 200 macrophages/coverslip. The concentration that inhibits 50% of growth (IC_50_) was calculated by dose-response and nonlinear regression analysis, using the GraphPad Prism^®^ 7.0 software.

### Cytotoxicity

Peritoneal macrophages (1 × 10^5^ cells/well) in complete RPMI medium were seeded in 96-well plates and incubated overnight at 37°C/5% CO_2_. After removing non-adherent cells, macrophages were treated with different concentrations of amentoflavone (0–22.3 µM) and incubated for another 72 h. Cell viability was determined using MTT colorimetric assay, as follows. After 4 h of incubation in the dark at 37°C with MTT, the culture supernatant was completely removed and 100 µl of DMSO added per well. For the complete dissolution of formazan crystals formed in viable cells, the plates were shaken for 15 min. The absorbance was measured at 570 nm using a spectrophotometer (EZ Read 400^®^, Biochrom). The CC_50_ value was determined by logarithmic regression analysis using GraphPad Prism^®^ 7.0. The selectivity index (SI) was calculated as macrophages CC_50_/intracellular amastigotes IC_50_.

### In Vivo Experimental Protocol

Female BALB/c mice (4–6 weeks-old) ranging from 20 to 25 g were randomly distributed into five groups. Animals of the groups 1 to 3 were subcutaneously (SC) infected with 1 × 10^4^ promastigote forms of *L. amazonensis* in the left hind footpad, while animals of the groups 4 and 5 were kept uninfected. As soon as the lesions appeared, on the 28^th^ day after the inoculum, all animals were weighed and the thickness of their footpads, right and left, was measured with a caliper (0.1 mm, Schnelltäster, HC Kroplin). Then, intralesional treatment (IL) with amentoflavone (0.5 mg/kg/dose) was initiated, through subcutaneous injection in the lesion site (groups 1 and 4). N-metil glucamine (64 mg Sb^5+^/kg/dose) was used as a positive control, and amentoflavone vehicle (10% Ethanol/10% Cremophor/1% DMSO/PBS) as a negative control of treatments (groups 3 and 5, respectively). All treatments were performed in five doses, administered 4 days apart. The animals’ weight and footpad thickness were monitored weekly. One week after the last treatment dose, animals were weighed and the lesion was measured just before being euthanized with Xylazine Hydrochloride (30 mg/kg, Syntec) associated with Ketamine Hydrochloride (300 mg/kg, Syntec). After euthanasia, tissue and blood samples were collected for subsequent analysis.

### Toxicology

Immediately after euthanasia, mice blood was collected (1 ml) *via* cardiac puncture and centrifuged to separate the serum. Sera were sent to the Animal Clinical Analysis Core Facilities of the Institute of Science and Technology in Biomodels (ICTB, Fiocruz RJ) to measurement of aspartate transaminase (AST), alanine transaminase (ALT), and creatinine in a Vitros 250 equipment (Ortho clinical - Jonhson & Jonhson).

### Parasite Load in the Lesion Site

Parasite load was estimated by a limiting dilution assay (LDA) (Cardoso et al., 2010). In brief, footpads were aseptically removed, weighed, and macerated in LIBHIT medium. Several sequential 1:2 dilutions were plated in 96-well “U” round-bottom plates, which were incubated at 26°C. Between the 5th and 10th day, the plates were analyzed by visualization in an inverted microscope (Axiovert 25^®^, Zeiss) to determine the last well containing at least one parasite in its promastigote form. The parasite load of footpads was calculated from the last dilution that contained parasites, divided by the weight of the respective organ. The results were expressed as the number of parasites per gram of tissue following the method described by Taswell (1984). The percentage of reduction in the parasite load was calculated considering the group treated with vehicles as having 100% of infection.

Quantitative PCR (qPCR) was used to confirm the parasite load in the lesion. After DNA extraction by a routine phenol-chloroform technique (Sambrook and Russell, 2006), 20 ng of total DNA were amplified using a specific primer for *Leishmania* sp. kDNA3 (forward 5’GGGTAGGGGCGTTCTGC3’, reverse 5’CCCGGCCTATTTTACACCAACC3’) (Weirather et al., 2011), as well as for the mouse β-actin endogenous control (forward 5’CTTGGCTGAACCATCAC3’, reverse 5’GGTCCTCATCGTTTAGCA3’) (Giulietti et al., 2001). Amplification was performed in a QuantStudio 3 equipment (Applied Biosystems) using GoTaq^®^ PCR Master Mix (Promega), with 250 nM of kDNA3 or 100 nM of β-actin primers per reaction. PCR conditions were as follows: hold at 95°C for 2 min, followed by 40 cycles of 95°C for 15 s and 62°C for 1 min followed by a dissociation curve. Standard curves were generated from 10-fold serial dilutions (100 ng–1 pg) of axenic *Leishmania* DNA and used to calculate parasite concentration in the samples.

### Histopathological Analysis

The lesion site, draining lymph nodes, spleen and liver were routinely processed for histopathological analysis. Five micrometers thick paraffin sections were obtained in a rotating microtome (HM 360, Microm) and stained using the hematoxylin-eosin (HE) or Giemsa techniques. Photomicrographs were obtained using a microscope (Axioplan 2, Zeiss) with image capture (AxioCam ERc 5s, Zeiss).

### Nitric Oxide Production by Peritoneal Macrophages

Peritoneal macrophages collected from all groups were plated in 96-well plates at a concentration of 5 × 10^5^ cells/well in RPMI complete medium and incubated for 48 h, at 37°C/5% CO_2_. Cells were stimulated with total *L. amazonensis* antigen (1 µg/ml) or Lipopolysaccharide (LPS, 1 µg/ml) (positive control). Unstimulated cells were maintained as negative control. Cell culture supernatants were harvested and used to evaluate nitric oxide production by the Griess reaction (Rizk et al., 2014).

### Local Expression of iNOS and Nrf2-Related Genes

Total RNA was extracted from mouse footpads using a standard TRI Reagent^®^ (Sigma-Aldrich) protocol, followed by DNAse treatment. RNA concentration and quality were determined by spectrophotometry (NanoDrop One, ThermoScientific). Complementary DNA (cDNA) was synthesized using iScript™ cDNA Synthesis kit (Bio-Rad) and 1 µg of total RNA, according to the manufacturer’s recommendations. Then, qPCR was performed on QuantStudio 3 equipment (Applied Biosystems). The sequences of the specific primers targeting mouse genes were *Nfe2l2* - *nuclear factor, erythroid derived 2, like 2, transcript variant 1* (Nrf2) (forward 5’TCACACGAGATGAGCTTAGGGCAA3’, reverse 5’TACAGTTCTGGGCGGCGACTTTAT3’), *Hmox1 - heme oxygenase 1* (HO-1) (forward 5’CCCAAAACTGGCCTGTAAAA 3’, reverse 5’CGTGGTCAGTCAACATGGAT3’), *L-ferritin* (forward 5’TTCCAGGATGTGCAGAAGCC3’, reverse 5’AAGAGGGCCTGATTCAGGTTC3’) and *Actb - actin, beta* (β-actin) (forward 5’AGCTGCGTTTTACACCCTTT3’, reverse 5’AAGCCATGCCAATGTTGTCT3’) (Tomiotto-Pellissier et al., 2018), as well as *Nos2 - nitric oxide synthase 2*, inducible, transcript variant 1 (iNOS) (foward 5’GGATCTTCCCAGGCAACCA3’, reverse 5’CAATCCACAACTCGCTCCAA3’) and *Rplp0 - ribosomal protein, large, P0* (Rplp0) (foward 5’GCCAGCTCAGAACACTGGTCTA3’, reverse 5’ATGCCCAAAGCCTGGAAGA3’) (Cardoso et al., 2020). The reaction mixtures contained 10 µl of GoTaq^®^ qPCR Master Mix (Promega), 20 ng of cDNA for Nrf2, HO-1 and β-actina targets, and 100 ng of cDNA for Rplp0 and iNOS targets; 100 nM of Nrf2, HO-1, ferritin and β-actin primers, 600 nM of iNOS primer and 900 nM of Rplp0 primer in a final volume of 20 µl. Cycling conditions were 2 min at 95°C, 40 cycles of 15 s at 95°C and 1 min at 60°C (HO-1, ferritin, β-actin) or 62°C (Nrf2, β-actin, Rplp0 and iNOS), followed by the dissociation curve. The results were analyzed with QuantStudio™ Design & Analysis software (Applied Biosystems). The relative quantification of mRNA of each gene was estimated by the 2^-ΔΔCT^ method, with the β-actin and Rplp0 mouse genes as endogenous control.

### ROS Measurement

The levels of intracellular ROS in macrophages infected and treated with amentoflavone were measured using the permeable dye 2’, 7’ - dichlorofluorescein diacetate (H_2_DCFDA). In the cell cytoplasm, this dye is deacetylated by cellular esterases and the compound formed oxidized by the ROS present, resulting in a highly fluorescent compound. Peritoneal macrophages obtained as described above were seeded in black 96-well plates at a density of 2 × 10^6^ cells/well. After 30 min, the plates were washed with PBS to remove non-adherent cells and the adhered cells kept at 34°C/5% CO_2_ overnight. Promastigote forms of *L. amazonensis* were added (10 parasites per cell) and the cells incubated for 5 h at 34°C. The cells were washed twice with PBS and the plate was kept an additional hour at 34°C before treatment. The concentrations of 1.15 μM (1/2 IC_50_), 2.3 μM (IC_50_), 4.6 μM (2× IC_50_), or 9.2 μM (4× IC_50_) of amentoflavone were added and the plates incubated for 24, 48 or 72 h. Antimycin B (10 mM) and glucose (60 mM) + glucose oxidase (20 units/ml) (G/GO) were used as positive controls. After the incubation times, medium was discarded and the macrophages washed once with Hank’s balanced salt solution (HBSS). Then, cells were incubated with H_2_DCFDA (20 μM) for 30 min at 37°C. Fluorescence was measured by fluorescence spectroscopy (SpectraMax M2, Molecular Devices), at 507 nm and 530 nm (excitation/emission wavelengths).

### Amentoflavone *In Silico* Toxicity Evaluation

To predict the toxicity of amentoflavone, the pkCSM tool was used (Pires et al., 2015). The SMILES (simplified molecular-input line-entry system) used for *in silico* analysis was as follows: C1=CC(=CC=C1C2=CC(=O)C3=C(O2)C(=C(C=C3O)O)C4=C(C=CC(=C4)C5=C C(=O)C6=C(C=C(C=C6O5)O)O)O)O.

### Ethics Statement

Female BALB/c mice (4–6 weeks-old) were obtained from the Institute of Science and Technology in Biomodels (ICTB, Fiocruz) and kept in an experimental animal facility with controlled room temperature, water, and food *ad libitum*. All procedures involving the use of animals were previously evaluated and approved by the Animal Use Ethics Committee of the Oswaldo Cruz Institute (CEUA/IOC), under licenses N°. L-053/2016 and N°. L-026/2019.

## Results

### Amentoflavone Was Active Against *L. amazonensis* Intracellular Amastigotes

**Figure 1:**
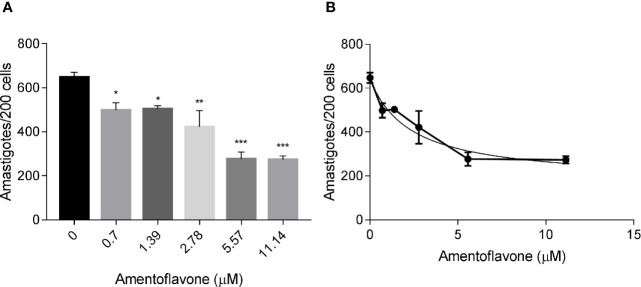
*In vitro* amentoflavone effect on macrophages infected with *L. amazonensis*. BALB/c mice peritoneal macrophages were infected with *L. amazonensis* promastigotes and treated with amentoflavone (0–11.14 μM) for 72 h. **(A)** Total intracellular amastigotes in 200 macrophages and **(B)** Dose-response curve of amentoflavone. Mock-treated infected cells were used as a control (0 μM). The columns represent the mean ± S.E.M. of quadruplicates. Significant difference (1-way ANOVA, followed by Tukey post-test) in relation to the mock-treated control *p < 0.05, **p < 0.01, ***p < 0.0001.

The treatment with amentoflavone reduced the amount of intracellular amastigotes in murine macrophages. The two highest tested concentrations (5.57 and 11.14 µM) reduced in 57% the total number of amastigotes, with no significant difference between them (t-test, p = 0.9244) (**Figure 1A**). The IC_50_ calculated from a non-linear regression curve was 2.3 ± 0.93 µM (**Figure 1B**).

### Amentoflavone Presented No Signs of Toxicity *In Silico*, *In Vitro* or *In Vivo*

To perform the *in silico* toxicity analysis of amentoflavone, we used the pkCSM tools (Pires et al., 2015). Amentoflavone showed absence of Ames toxicity, hepatotoxicity, skin sensitization, Minnow toxicity, and non-inhibitor of hERG I (**Supplementary Table I**), suggesting that amentoflavone is safe. In fact, amentoflavone was not cytotoxic to peritoneal macrophages at the tested concentrations for a period of 72 h. The viability of cells treated with the highest tested concentration (22.3 µM) was not significantly different from the mock-treated control cells. The selectivity index *in vitro* was estimated to be greater than 10, but it could not be calculated since the higher concentration essayed was not toxic to cells. When the drug was intralesionally injected in BALB/c infected mice, there was no weight loss and the survival rate was 100% throughout the treatment. Intralesional treatment with amentoflavone did not show hepatic or renal toxicity since no alterations were observed in the biochemical markers ALT, AST and creatinine. Similar results were observed in animals treated with the reference drug (Glucantime^®^) and in mock-treated controls.

### Amentoflavone Reduced Parasitic Burden in *L. amazonensis*-Infected Mice

**Figure 2:**
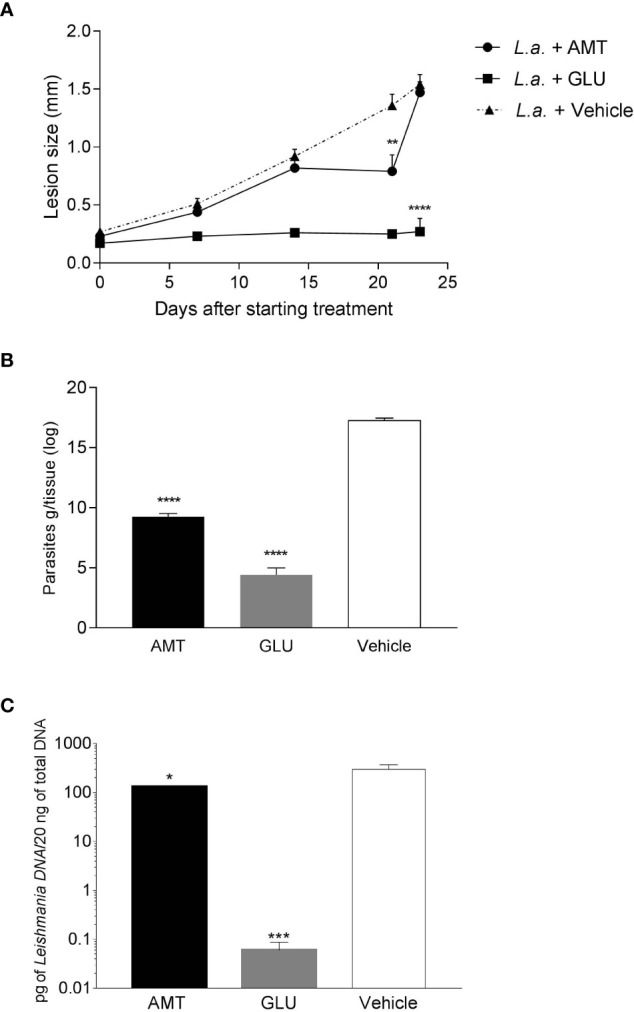
Effect of intralesional amentoflavone treatment on *L. amazonensis*-infected BALB/c mice. **(A)** Lesion size kinetics. The size of the lesions was monitored weekly from the beginning of treatment until 1 week after the last treatment dose by measuring the footpad with a caliper, with 0.1 mm sensitivity. The data shown represents the average of the difference between the infected footpad and the uninfected contralateral footpad ± S.E.M. of 10 animals per group. **(B)** Parasite load estimated by limiting dilution assay. **(C)** Parasite load estimated by qPCR. Points and bars represent the mean ± S.E.M. of five animals per group. *p < 0.05, **p < 0.01, ***p < 0.001, ****p < 0.0001: significant difference in relation to the mock-treated control (1-way ANOVA, followed by Tukey post-test). *L.a.*, Animals infected with *L. amazonensis*; AMT, Amentoflavone; GLU, Glucantime.

Intralesional treatment with amentoflavone (0.05 mg/kg/dose every 4 days in a total of 5 doses) led to a significant reduction (p = 0.0014) in the size of the lesion just after the last dose of subcutaneous treatment, 21 days after the start of treatment, in comparison to the mock-treated group. Nevertheless, after the end of treatment, lesions regained their growth, reaching the same size as the control, one week later. The group that received intralesional Glucantime^®^ maintained the same lesion size throughout the treatment, reaching the end of it with a significantly smaller lesion than the control group treated with vehicle (p < 0.0001) (**Figure 2A**).

Seven days after the last dose of intralesional treatment, animals were euthanized and the parasite load at the lesion site measured by a limiting dilution assay (LDA) and quantitative PCR (qPCR). Amentoflavone treatment significantly reduced the number of viable parasites by 46.3% and parasite DNA by 56.1% in the footpad, when compared to vehicle-treated animals (p < 0.0001 and p = 0.0292, for ADL and qPCR, respectively). Glucantime^®^ induced a strong reduction in the parasite load (74.4% and 99.9%, for ADL and qPCR, respectively) as compared to mock-treated animals (**Figures 2B, C** and **Supplementary Table II**). The two techniques used to calculate parasite load presented a positive correlation (r = 0.825 on a Spearman test; p = 0.0003).

### Histological Findings

**Figure 3:**
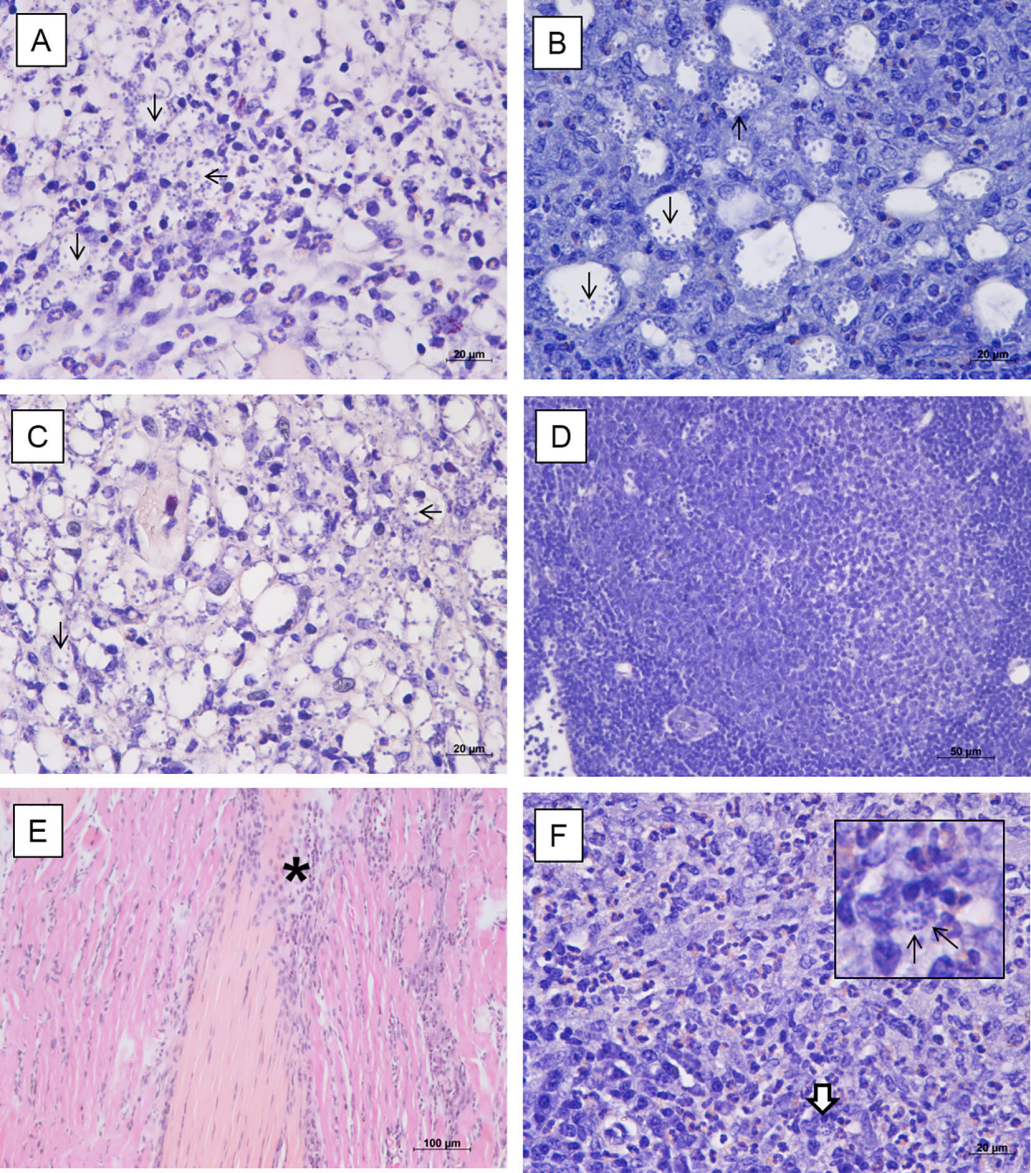
Histopathological analysis of BALB/c mice treated intralesionally. Photomicrographs of the footpad and lymph nodes of BALB/c mice infected with *L. amazonensis* and treated with vehicle **(A, B)**, amentoflavone 0.5 mg/kg/dose **(C, D)** or Glucantime 64 mg Sb^5+^/kg/dose **(E, F)**. **(A)** Inflammatory infiltrate with polymorphonuclear cells and intense parasitism (black arrows) in the footpad (hematoxylin and eosin); **(B)** Infected macrophages (black arrows) in the lymph node (Giemsa). **(C)** Amastigotes in footpad (black arrow) (hematoxylin and eosin). **(D)** Hyperplastic germinal center in the lymph node with follicular hyperplasia (Giemsa). **(E)** Connective tissue thickening among the muscles and inflammatory infiltrate (asterisk) in the footpad (hematoxylin and eosin). **(F)** Polymorphonuclear cells (mainly eosinophils), parasitism (detail, white arrow) in the lymph node (Giemsa). Representative images (2 animals/group).

Histological evaluation of footpad, draining popliteal lymph node, liver and spleen of non-infected animals did not show evident histopathological alterations. However, discrete foci of inflammatory infiltrate were observed in the footpad, with the presence of mast cells and a slight increase in collagen fibers, possibly due to the trauma of the subcutaneous injections of the vehicle. On the other hand, footpads of the infected mock-treated animals showed an intense parasitism among the muscle fibers; thinning of the conjunctiva between the superficial dermis and the epidermis; mast cells in the middle of the dermis and the first muscle layer; macrophages intensely parasitized and presence of an inflammatory infiltrate predominantly composed of polymorphonuclear cells (**Figure 3A**). The lymph nodes showed intense parasitism and presence of giant cells (**Figure 3B**). No histopathological alterations were observed in the liver and spleen of these animals. The infected animals treated with 0.05 mg/kg/dose of amentoflavone also showed moderate to intense parasitism in the footpad, with a similar inflammatory infiltrate (**Figure 3C**). The lymph nodes presented amastigotes and strong lymphoid activation and proliferation of germinal centers, with follicular hiperplasia (**Figure 3D**). The liver and spleen showed no parasitism, with the later showing a greater lymphoid activation and several of germinal centers in comparison to the infected mock-treated group. Footpad of infected animals treated with 64 mg Sb^5+^/kg/dose of Glucantime^®^ showed low parasitism; fibrosis foci; presence of fibroblasts, macrophages, mast cells, and some eosinophils (**Figure 3E**). The lymph node showed low parasitism; presence of giant cells, as well as many polymorphonuclear cells and some mast cells (**Figure 3F**). The spleen, on the other hand, did not show parasitism. No changes were found in the liver of these animals.

### Amentoflavone Exerts Both Antioxidative and Prooxidative Responses

**Figure 4:**
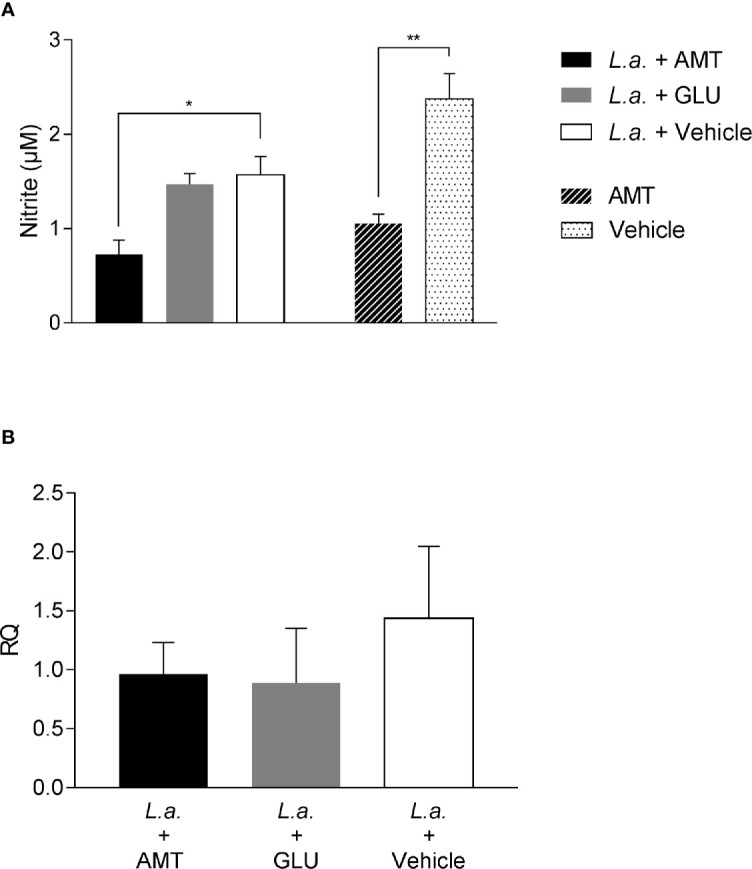
Evaluation of NO production and iNOS expression on amentoflavone-treated mice **(A)** Nitrite quantification in peritoneal macrophages isolated from *L. amazonensis*-infected BALB/c, treated or not with amentoflavone intralesionally. Macrophages were stimulated with total *L. amazonensis* antigen (1 µg/µl) and the amount of nitrite in the cell culture supernatant was measured by the Griess reaction. The bars represent the mean ± S.E.M. of five animals per group. *p < 0.05, **p < 0.01 (Test t). **(B)** Relative quantification of iNOS mRNA in *L. amazonensis*-infected BALB/c mouse footpads treated or not with amentoflavone or Glucantime, intralesionally. Expression was estimated by 2-ΔΔCT method, using Rplp0 as a reference gene. The bars represent the mean ± S.E.M. of three animals per group. *L.a.*, Animals infected with *L. amazonensis*; AMT, Amentoflavone; GLU, Glucantime.

To evaluate whether NO production could be associated with amentoflavone mechanism of action, peritoneal macrophages from BALB/c mice, infected or not with *L. amazonensis* and treated intralesionally with amentoflavone or Glucantime were stimulated *in vitro* with soluble antigens of *L. amazonensis*, LPS (positive control) or medium (negative control). As expected, LPS stimulated-cells produced NO, whereas non-stimulated cells did not. *Leishmania* antigens were able to stimulate NO production, but peritoneal macrophages taken from mice treated with amentoflavone produced significantly lower NO than cells from mock-treated animals. This decrease occurred in cells derived from both infected and non-infected animals (p = 0.0270 and p = 0.0035, respectively) (**Figure 4A**). In addition, when iNOS expression in the lesion was evaluated by RT-qPCR, although no significant difference was observed, treated animals presented slightly lower iNOS expression than infected mock-treated animals (**Figure 4B**).

**Figure 5:**
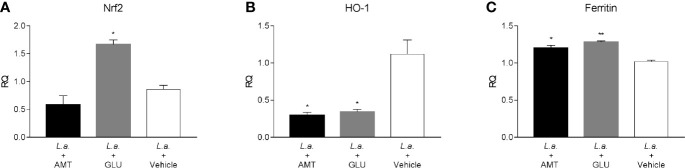
Relative quantification of Nrf2-related genes in *L. amazonensis*-infected BALB/c mice footpads treated or not with amentoflavone or Glucantime, intralesionally. RT-qPCR analyses were performed to quantify the expression of Nrf2 **(A)**, HO-1 **(B),** and ferritin **(C)** genes. Expression was estimated by 2-^ΔΔCT^ method, using Actb as a reference gene. The bars represent the mean ± S.E.M. of three animals per group. Significant differences (1-way ANOVA, followed by Tukey post-test); *p < 0.05; **p < 0.01 in relation to the mock-treated control. Nrf2, *nuclear factor, erythroid derived 2, like 2*; HO-1, *heme oxygenase 1*; Feritin, *L-ferritin*. Actb, *actin beta*.

Recently, amentoflavone has been reported as a molecule capable of triggering the Nrf2 activation pathway, a gene responsible for triggering antioxidant responses in the cell (Chen et al., 2018; Wahyudi et al., 2018). Several studies point out to Nrf2 as responsible for the antioxidant effect of leishmanicidal compounds (Tomiotto-Pellissier et al., 2018; Cataneo et al., 2019; Miranda-Sapla et al., 2019). Therefore, the expression of Nrf2, as well as HO-1 and Ferritin genes, both regulated by Nrf2, were investigated in the footpad of animals infected with *L. amazonensis* and treated with amentoflavone intralesionally. Treatment with amentoflavone did not alter the expression of Nrf2, which was increased in Glucantime^®^-treated footpads (p = 0.0172). Although Nrf2 expression was not altered at that time point, HO-1 expression in the amentoflavone-treated footpad was significantly reduced (p = 0.0120), as well as Glucantime^®^-treated footpad (p = 0.0236). The ferritin gene expression was increased both in amentoflavone and Glucantime^®^-treated groups when compared to the control group (p = 0.0168 and p = 0.0042, respectively) (**Figure 5**).

**Figure 6:**
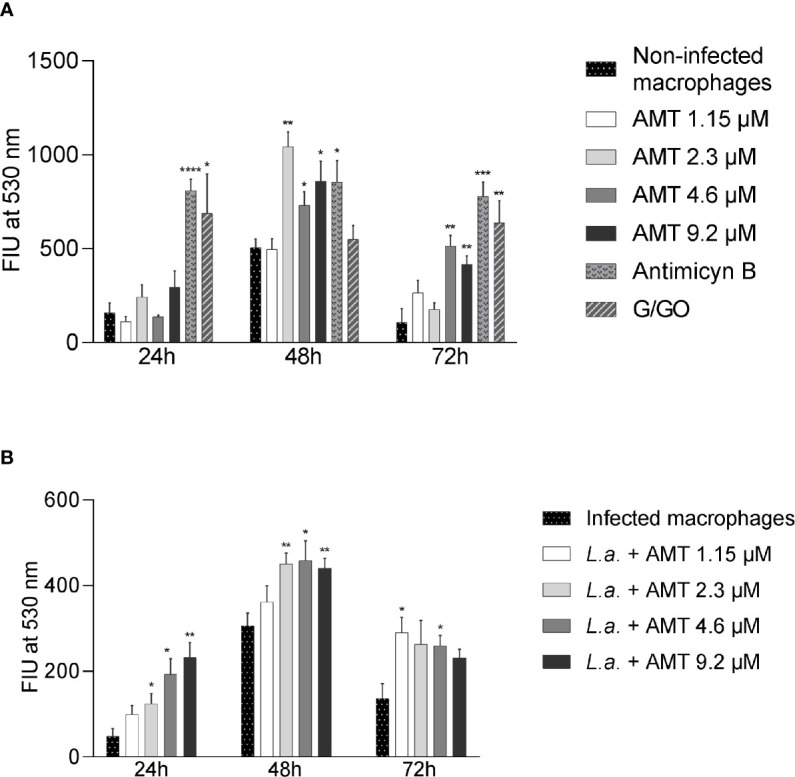
Amentoflavone concentration influence on the ROS production in peritoneal macrophages. Murine peritoneal macrophages (2 × 10^6^) were treated with different concentrations of amentoflavone. **(A)** Uninfected macrophages treated with amentoflavone in concentrations of 1.15 μM (1/2 IC_50_), 2.3 μM (IC_50_), 4.6 μM (2× IC_50_) or 9.2 μM (4× IC_50_) for 24, 48, and 72 h. Mock-treated macrophages were used as controls. Antimycin B and Glucose/Glucose oxidase (G/GO) were used as a positive control. **(B)** Macrophages infected with *L. amazonensis* and treated with different concentrations of amentoflavone for 24, 48, or 72 h. Infected and mock-treated macrophages were used as controls. The generation of ROS was measured using the fluorescent indicator H_2_DCFDA. The data were expressed in units of fluorescence intensity (FIU). Significant differences in relation to the control (t test): *p < 0.05, **p < 0.01, ***p < 0.001 and ****p < 0.0001. AMT, Amentoflavone; *L.a.*, *L. amazonensis*.

Since amentoflavone’s mechanism of action could not be explained by an increase of NO production, the generation of ROS was evaluated *in vitro*. The ROS production was measured in amentoflavone-treated and non-infected peritoneal macrophages obtained from normal BALB/c mice (**Figure 6A**). After 24 h of treatment, amentoflavone did not alter the production of ROS. However, after 48 h of treatment, this production was significantly higher in cells treated with 2.3 µM (IC_50_) (p = 0.0009) or more of amentoflavone (4.6 µM: p = 0.0393, 9.2 µM: p = 0.0280). The increase in ROS was still noticed after 72 h of treatment with the two highest concentrations (4.6µM: p = 0.0022, 9.2 µM: p = 0.0069), but not in cells treated with 2.3 µM, which had the highest production in 48 h. Antimycin B and Glucose/Glucose oxidase, both used as positive controls, presented significantly higher production than non-treated normal cells at all times. When ROS production was evaluated in *L. amazonensis*-infected macrophages, amentoflavone increased ROS production at 2.3 µM or higher at 24 and 48 h, decreasing at 72 h, when the lower concentration 1.15 µM increased the ROS production in relation to mock-treat infected cells (**Figure 6**).

## Discussion

Amentoflavone action on *Leishmania* parasites has been controversial. Weniger and collaborators (Weniger et al., 2006) found no activity against *L. donovani* axenic amastigotes, but our group have shown that amentoflavone can disrupt the membrane potential of mitochondria and effectively kill *L. amazonensis* promastigotes (manuscript in preparation). When amentoflavone was tested on intracellular amastigote forms of *L. amazonensis*, it showed antileishmanial activity and a good selectivity index (SI) (Oubada et al., 2014). Corroborating these data, amentoflavone isolated from *S. sellowii* also showed potential antileishmanial activity on this same *Leishmania* species and cellular form (Rizk et al., 2014). In this work, we evaluated the activity of commercially acquired amentoflavone on these intracellular amastigote forms, reaching an IC_50_ value of 2.3 μM, which confirms the antileishmanial activity of this biflavonoid. In addition, *in silico* data suggested that amentoflavone presents low toxicity. These data was corroborated by an experimental SI greater than 10, pointing out to the safe use of amentoflavone as a chemotherapeutic agent.

In order to evaluate the effect of amentoflavone treatment on cutaneous leishmaniasis *in vivo*, BALB/c mice were infected with promastigote forms of *L. amazonensis* and treated *via* intralesional injection. Once the oral route does not seem to be the preferred form of amentoflavone administration due to its low bioavailability (Liao et al., 2015), in this work a local application was chosen, as already used in the clinic for cutaneous leishmaniasis treatment, in order to prevent systemic metabolism and increase bioavailability at the lesion site. Animals treated either with amentoflavone or Glucantime^®^ showed no signs of toxicity, including no alteration on ALT, AST, and creatinine levels, no decreased body weight or death, corroborating data from the literature (Lee et al., 2018; Liu and Yu, 2018; Shen et al., 2018; Tsai et al., 2018).

Throughout the intralesional treatment of *L. amazonensis-*infected mice, the amentoflavone-treated group showed an increase in the thickness of the lesion similar to that observed in the mock-treated group. It is interesting to note that, just after the last dose of treatment (21 days post-treatment), amentoflavone-treated mice had their lesion size reduced when compared to the mock-treated group. However, 7 days later, at the end of the experimental protocol, the increase in lesion thickness caught up to the average thickness of the mock-treated group. Although the amentoflavone treatment has not been effective in maintaining the lesions reduction, both LDA, which quantifies only viable parasites, and the real-time PCR, which quantifies parasite DNA, showed a reduction in the parasite load in the lesion of the group treated with amentoflavone (0.05 mg/kg/dose). However, as this reduction was not absolute (between 45% and 56%), the lesion remained active after the last dose and regained its growth. It is possible that the use of higher dosages and/or a longer therapeutic regimen might increase this activity *in vivo*.

N-metil glucamine treatment maintained the initial thickness of the lesion until the end of the experiment. In fact, the reference drug reduced the parasite load by 74% to 99% compared to mock-treated animals. Despite reducing the amount of parasitic DNA present in infected footpads by almost 100%, treatment with Glucantime did not result in a sterile cure. In fact, studies show that treatment with intralesional Glucantime does not completely eradicate the parasite in BALB/c mice (de Paladi et al., 2012; García et al., 2017; Tamargo et al., 2017). The dose of Glucantime used in our study was based on a guidance of the Ministry of Health in Brazil, which predicts the administration of intralesional non-dilluted Glucantime^®^ through infiltration at the base of the skin lesion, until the area swells (Brasil, 2017). As a result, the volume may vary depending on the affected region and the size of the lesion. In our experimental protocol, for standardization purposes, we chose to apply 0.02 ml of Glucantime^®^ per mouse, without previous dilution, reaching a dose of 64 mg of Sb^5+^/dose, which was able to reduce almost completely the parasitic burden in the footpads after five subcutaneous injections. Our results, combined with data in the literature, including studies with humans, lead us to reinforce the effectiveness of the intralesional use of Glucantime in the treatment of localized skin lesions. The intralesional use of amentoflavone has shown to have potential in the treatment of the cutaneous lesion caused by *L. amazonensis* in BALB/c mice. However, the use of a higher dosage may result in an increased effect *in vivo*. The difficulty of solubilizing higher concentrations of the compound in a vehicle that can be applied subcutaneously was a challenge. The difficulty in dissolving amentoflavone in water and organic solvents is known (Feng et al., 2020). In order to improve solubility in liquid solvents, there are several pharmacotechnical possibilities. Recently, micelles and nanomicelles have been used to improve the solubility and bioavailability of amentoflavone (Zhang et al., 2019; Feng et al., 2020). Also, the use of amorphous solid dispersion contributed to the increase in solubility, dissolution and oral bioavailability of amentoflavone, promoting the antitumor effect in rats (Chen et al., 2020). Therefore, it is suggested that the pharmacotechnical improvement of amentoflavone may increase its antileishmanial activity *in vivo*.

It is known that the interactions that initially occur between the *Leishmania* parasite and the immune cells can shape the macrophages response and the type of adaptive immune response that is being induced. The first interactions between the protozoan and eosinophils and mast cells influence the response of macrophages to infection and the development of the adaptive immune response, thus determining the final result of the infection (Rodríguez and Wilson, 2014). The histopathological analysis of our experimental protocol showed the presence of these cells in all infected groups. The decrease in parasitism with the presence of mast cells in the footpad of animals treated with Glucantime may be associated with the remodeling of the tissue injured by the parasite. In dogs naturally infected with *L. infantum*, the low parasitism and the presence of few clinical signs were associated with a higher density of mast cells and deposition of type III collagen in tissues of oligosymptomatic animals (Cardoso et al., 2017). The findings observed in histopathology regarding the presence of parasites were compatible with the parasite load quantification, both by LDA and by qPCR. Amentoflavone treated group presented follicular hyperplasia, which is often seen during leishmaniasis and argues for the effective activation of the immune response (Corbett et al., 1992). The group treated with Glucantime^®^ had, in fact, lower amastigotes colonization, when compared to the other groups. It is interesting to highlight the persistence of the protozoan in the lymph nodes of this group after lesion reduction, which might serve as a reservoir of the parasite. The persistence of parasites in the lymph nodes was also shown by our group after clinical cure of the lesions in C3H/He *L. amazonensis*-infected mice (Cardoso et al., 2010; de Souza et al., 2018). These findings reinforce the need to consider this organ as a reservoir of the protozoan even after the clinical cure of the lesion.

Several cellular processes begin after the activation of macrophages infected by the protozoan *Leishmania*, including liposomal degradation of enzymes, NO and reactive oxygen species production (Van Assche et al., 2011). The oxidative attack is the main weapon used by cells against invading pathogens (Paiva and Bozza, 2014).

Bearing in mind that NO is an important inflammatory mediator in infection by *Leishmania* sp. (Liew et al., 1990; Stenger et al., 1994; Lima-Junior et al., 2013), the analysis of its production also becomes an interesting strategy in the investigation of the immunological factors related to the treatment of the cutaneous lesion generated by *L. amazonensis*. Our previous work showed that amentoflavone treatment reduces NO production in macrophages infected by *L. amazonensis* (Rizk et al., 2014). Similarly, the results obtained in the present work showed that amentoflavone treatment reduced the NO production in infected macrophages obtained from both infected and uninfected animals. In addition, amentoflavone treatment was also able to reduce the iNOS expression in the lesions of infected mice, reinforcing the role of amentoflavone in the reduction of nitric oxide. These results suggest that this inflammatory mediator is not the mechanism of parasitic death and that the antileishmanial activity of amentoflavone would not be directly related to the reduction of this free radical, but with its antioxidant capacity (Rizk et al., 2014). In fact, parasite death is not always linked to the production of NO. Studies show that only NO production is not enough to control the infection (Mukbel et al., 2007; Santos-Pereira et al., 2019).

The production of reactive oxygen species (ROS) is another mechanism that can interfere with the elimination of microbes (Paiva and Bozza, 2014). In the present study, amentoflavone treatment was pro-oxidant *in vitro*, inducing an increase of ROS in peritoneal macrophages. This increase was independent of intracellular infection, although the presence of the parasite seems to anticipate this event, as seen at 24 h after treatment. In fact, it has been reported that amentoflavone induces the formation of hydroxyl radicals and thus acts synergistically with antibiotics to exert a microbicidal effect on gram-negative bacteria (Hwang et al., 2013). The generation of these hydroxyl radicals has also been associated with mitochondrial dysfunction and apoptosis in *Candida albicans* cells (Hwang et al., 2012) and in breast cancer cells (Pei et al., 2012). Oxidative stress generated by the release of ROS has already been reported as a leishmanicidal mechanism in other flavonoids (Fonseca-Silva et al., 2013; Cataneo et al., 2019) and, therefore, we suggest that this is one of the means by which amentoflavone exert its antileishmanial activity.

The transcription factor Nrf2 is responsible for the balance between the antioxidant and pro-inflammatory profiles and has attracted attention in studies that investigate the immune system in the modulation of infectious diseases (Vivarini and Lopes, 2020). *In vitro* studies have attributed the positive regulation of Nrf2 to the activity of leishmanicidal compounds (Tomiotto-Pellissier et al., 2018; Cataneo et al., 2019; Miranda-Sapla et al., 2019). In recent years, amentoflavone has been associated with the reduction of oxidative stress *in vivo* through the regulation of Nrf2. In a model of Alzheimer’s disease in rats, amentoflavone exerted a neuroprotective effect, attributed to the ability to decrease oxidative stress by inducing Nrf2 (Chen et al., 2018). In our model, amentoflavone did not alter Nrf2 expression. Nonetheless, HO-1 was downregulated. The level of expression of this gene can be related to the reduction of parasite load in the lesion, since it’s known that *Leishmania* protozoan induces the expression of the human heme oxygenase gene (HMOX1) due to its lipophosphoglycans (LPG) (Luz et al., 2012; De Menezes et al., 2019; Saha et al., 2019). *L. amazonensis* induces higher amounts of this antioxidant enzyme than *L. major*, which may have a role in the outcome of the different clinical forms of the disease (De Menezes et al., 2019). It was expected that the decrease in HO-1 would be accompanied by a decrease in the iron-sequestering protein, given the metabolic relationship between the two. However, we found an increase in the expression of ferritin in both amentoflavone and Glucantime^®^ treated mice. It is possible that parasite death led to an increase of free iron inside the cell, which is known to regulate ferritin synthesis without the participation of heme (Eisenstein et al., 1991).

The increase in ROS associated with high concentrations of amentoflavone *in vitro* together with the reduction of NO *in vivo*, led us to investigate the role of this biflavonoid in the balance between inflammatory and anti-inflammatory patterns at the infection site, known to be determinant in the development of cutaneous leishmaniasis. In addition, the alteration of the expression of antioxidant genes, associated with the reduction of parasites in the lesion reinforces the need for studies that deepen the role of these mechanisms in the pathogenesis of leishmaniasis. Therefore, we demonstrate that amentoflavone has an antileishmanial effect both *in vitro* and *in vivo*, acting on the balance of the inflammatory response, and may represent an ally in the control of the leishmaniotic lesion.

## Data Availability Statement

The original contributions presented in the study are included in the article/**Supplementary Material**. Further inquiries can be directed to the corresponding author.

## Ethics Statement

The animal study was reviewed and approved by Comissão de Ética no Uso de Animais do Instituto Oswaldo Cruz (CEUA-IOC).

## Author Contributions

Study design: YR, CA, CC, and KC. Data collection and analysis: YR, FC, CS, LG, TZV, and MP-M. Funding acquisition: KC and EA. Investigation: YR. Methodology: FC, CS, LG, SS-P, DH, and MP-M. Project administration and supervision: KC. Writing—original draft: YR, TZV, and KC. Writing—review and editing: FC, TZV, CA, EA, and KC. All authors contributed to the article and approved the submitted version.

## Funding

This work was supported by a grant from Instituto Oswaldo Cruz/Fundação Oswaldo Cruz, Fundação Carlos Chagas Filho de Amparo à Pesquisa do Estado do Rio de Janeiro (FAPERJ) [E-26/203.347/2017, E-26/010.101082/2018 and E-26/010.001759/2019]. EA and MP-M are recipients of research scholarships from Conselho Nacional de Desenvolvimento Científico e Tecnológico (CNPq) (CNPq processes 304904/2016-3 and 313520-2018-6, respectively). YR is the recipient of a research scholarship from Coordenação de Aperfeiçoamento de Pessoal de Nível Superior, Brasil (CAPES) [Finance Code 001]. The funders had no role in study design, data collection and analysis, decision to publish, or preparation of the manuscript.

## Conflict of Interest

The authors declare that the research was conducted in the absence of any commercial or financial relationships that could be construed as a potential conflict of interest.
