# Supplementary material for: Amentoflavone as an Ally in the Treatment of Cutaneous Leishmaniasis: Analysis of Its Antioxidant/Prooxidant Mechanisms
Source: Front Cell Infect Microbiol. 2021 Feb 25;11:615814. doi: 10.3389/fcimb.2021.615814 (PMC7950538; doi:10.3389/fcimb.2021.615814)
Supplement: Supplementary file 1 [file Table_1.docx]

**Supplementary Table 1.** Amentoflavone *in silico* toxicity predictions.

| **Model Name** | **Predicted Value** |
| --- | --- |
| AMES toxicity | No |
| Maximal tolerated dose (human) | 2.74 mg/kg/day |
| hERG I inhibitor | No |
| hERG II inhibitor | Yes |
| Oral Rat Acute Toxicity (LD50) | 2.527 mol/kg |
| Oral Rat Chronic Toxicity (LOAEL) | 3.73 g/kg bw/day |
| Hepatotoxicity | No |
| Skin Sensitisation | No |
| *T. pyriformis* toxicity | 1.93 mg/L |
| Minnow toxicity | 484.2 mM |

*T. pyriformis* toxicity = value greater than 0.32 mg/L is considerate toxic;

Minnow toxicity = value below 0.5 mM are regarded as high acute toxicity.

bw = body weight
