# Supplementary material for: Amentoflavone as an Ally in the Treatment of Cutaneous Leishmaniasis: Analysis of Its Antioxidant/Prooxidant Mechanisms
Source: Front Cell Infect Microbiol. 2021 Feb 25;11:615814. doi: 10.3389/fcimb.2021.615814 (PMC7950538; doi:10.3389/fcimb.2021.615814)
Supplement: Supplementary file 2 [file Table_2.docx]

**Supplementary Table 2.** Parasitic load quantification by limiting dilution assay in BALB/c mice footpad infected with *L. amazonensis*, one week after the end of intralesional treatment.

| **Treatment** | **Difference from control (%)** | | **Average number of parasites/g tissue*** |
| --- | --- | --- | --- |
|  | **Limiting Dilution Assay** | **qPCR** |  |
| Amentoflavone | - 46.3 | - 56.1% | 3.39x10^9^ |
| Glucantime | - 74.4 | - 99.9% | 3.03x10^5^ |
| Vehicle | - | - | 2.24x10^17^ |

*Limiting Dilution Assay
